# Sequence variability is correlated with weak immunogenicity in *Streptococcus pyogenes* M protein

**DOI:** 10.1002/mbo3.278

**Published:** 2015-07-15

**Authors:** Jonas Lannergård, Bodil M Kristensen, Mattias C U Gustafsson, Jenny J Persson, Anna Norrby-Teglund, Margaretha Stålhammar-Carlemalm, Gunnar Lindahl

**Affiliations:** 1 Department of Laboratory Medicine, Lund UniversityLund, Sweden; 2 Department of Veterinary Disease Biology, University of CopenhagenFrederiksberg C, Denmark; 3 Department of Experimental Medical Science, Lund UniversityLund, Sweden; 4 Center for Infectious Medicine, Karolinska Institutet, Huddinge University HospitalStockholm, Sweden

**Keywords:** Antibody escape, group A *Streptococcus*, immunodominance, immunogenicity, protease sensitivity, sequence divergence

## Abstract

The M protein of *Streptococcus pyogenes*, a major bacterial virulence factor, has an amino-terminal hypervariable region (HVR) that is a target for type-specific protective antibodies. Intriguingly, the HVR elicits a weak antibody response, indicating that it escapes host immunity by two mechanisms, sequence variability and weak immunogenicity. However, the properties influencing the immunogenicity of regions in an M protein remain poorly understood. Here, we studied the antibody response to different regions of the classical M1 and M5 proteins, in which not only the HVR but also the adjacent fibrinogen-binding B repeat region exhibits extensive sequence divergence. Analysis of antisera from *S. pyogenes*-infected patients, infected mice, and immunized mice showed that both the HVR and the B repeat region elicited weak antibody responses, while the conserved carboxy-terminal part was immunodominant. Thus, we identified a correlation between sequence variability and weak immunogenicity for M protein regions. A potential explanation for the weak immunogenicity was provided by the demonstration that protease digestion selectively eliminated the HVR-B part from whole M protein-expressing bacteria. These data support a coherent model, in which the entire variable HVR-B part evades antibody attack, not only by sequence variability but also by weak immunogenicity resulting from protease attack.

## Introduction

The gram-positive bacterium *Streptococcus pyogenes* (group A *Streptococcus*) is a human-specific pathogen responsible for mild throat and skin infections and for life-threatening conditions resulting in ∼500,000 deaths annually (Carapetis et al. 2005). The most studied virulence factor of this pathogen is the polymorphic and surface-localized M protein, a dimeric coiled-coil molecule that inhibits phagocytosis and contributes to virulence also by other mechanisms (Fischetti 1989; Waldemarsson et al. 2009). This fibrillar protein has an amino-terminal and wall-distal hypervariable region (HVR), which exhibits extreme sequence variability among strains but not within a strain, allowing the identification of ∼200 M (or *emm*) types (Steer et al. 2009). The HVR has attracted much attention, because it is a target for type-specific protective antibodies (Abs), is used for typing and classification, commonly binds a host ligand, and is evaluated as a vaccine component (Fischetti 1989; Morfeldt et al. 2001; Persson et al. 2006; Dale et al. 2011; Gustafsson et al. 2013; Sanderson-Smith et al. 2014).

We previously presented evidence that the HVR of an M protein elicits a much weaker antibody response than the remaining part of the protein, although the HVR is a key target for protective Abs (Lannergård et al. 2011). This property may seem paradoxical, but should be advantageous to the bacterium, by allowing it to escape anti-HVR Abs by two mechanisms, which act at different stages of an infection. While sequence variability causes antigenic variation that favors the establishment of an infection, a weak Ab response will delay the appearance of protective immunity in an infected host, thereby prolonging the infection.

**Figure 1:**
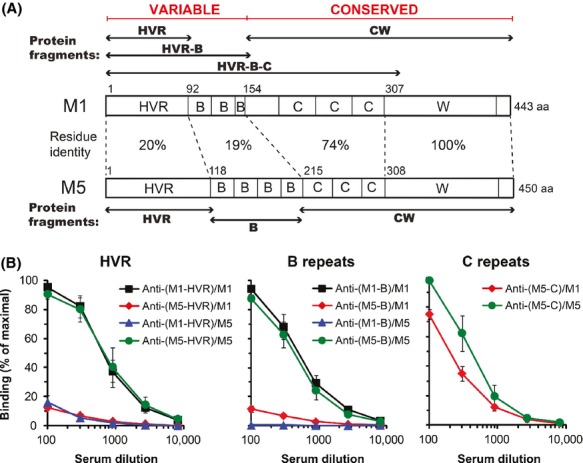
Schematic representations of the M1 and M5 proteins and analysis of cross-reactivity. (A) The M1 and M5 proteins have similar overall domain arrangement, with an amino-terminal HVR, a fibrinogen-binding B repeat region, a C repeat region and a wall-associated W region. Extensive sequence divergence characterizes not only the HVRs but also the B repeat regions, implying that M1 and M5 can be divided into a variable and a conserved part, as shown. Recombinant fragments of M1 and M5 used in this report are indicated. (B) Analysis showing virtual lack of cross-reactivity between the HVRs or between the B repeat regions, while the C repeat regions cross-react. Rabbit antisera, directed against the region in M1 or M5 indicated at the top of each panel, were used to test reactivity with intact M1 or M5 immobilized in microtiter wells. For example, anti-(M1-HVR)/M5 indicates the reactivity of anti-(M1-HVR) with immobilized intact M5. Because an antiserum raised against the C repeats of M1 was not available, analysis of cross-reactivity between the C repeats was only performed with anti-(M5-C). Bound rabbit Abs were detected with radiolabeled protein G. A low background binding observed with preimmune rabbit serum has been subtracted. Data in panel B show mean values ± SD and each analysis was performed three times.

The intriguing finding that the HVR of an M protein is weakly immunogenic prompted us to perform the studies reported here, aimed at systematically analyzing the Ab response to different regions of an M protein and at studying the molecular basis for weak immunogenicity. Studies of two different M proteins was deemed important, to ensure that any findings would not reflect properties unique to one protein. As in our previous study, we employed the M1 and M5 proteins, two classical M proteins that have been epidemiologically associated with life-threatening invasive infections and rheumatic fever, respectively, the most serious diseases caused by *S. pyogenes* (Carapetis et al. 2005). These M proteins have similar overall structure, with an HVR followed by a fibrinogen (Fg)-binding B repeat region and a carboxy-terminal part comprising C repeats and a wall-associated W region (Fig.1A) (McMillan et al. 2013). The sequences of the B repeat regions of M1 and M5 are as divergent as those of the HVRs, implying that M1 and M5 can be divided into a variable HVR-B part and a relatively conserved CW part (Fig.1A). This observation, and evidence from the M5 system that each of the HVR and B regions contributes to virulence (Waldemarsson et al. 2009), suggested that both of these regions might escape Ab attack by similar mechanisms. We therefore hypothesized that weak immunogenicity characterizes not only the HVR but also the B repeats, although it has been reported that the B repeats of an M protein are immunodominant (Fischetti and Windels 1988).

The studies of M1 and M5 described here show that both the HVR and the B repeat region indeed are weakly immunogenic, whereas the carboxy-terminal part is immunodominant. Thus, we identified a correlation between sequence variability and weak immunogenicity for M protein regions. A possible molecular explanation for the weak immunogenicity was provided by the demonstration that proteases selectively eliminated the HVR-B part from bacteria-bound M protein. On the basis of these data, we propose that the entire variable HVR-B part evades Ab attack not only through sequence variability, but also through weak immunogenicity resulting from protease attack.

## Experimental Procedures

### Bacterial strains and culture conditions

The reference *S. pyogenes* strains used here, the M1 strain SF370 (Ferretti et al. 2001) and the M5 strain Manfredo (Miller et al. 1988), were obtained from Dr M. Kehoe. The M1 strain 5448 and an SpeB-negative mutant of that strain (5448Δ*speB*) were from Dr V. Nizet (Kansal et al. 2003). The M1 strain MC25, which secretes an M1 protein lacking the most carboxy-terminal part, was from Dr M. Collin (Collin and Olsén 2000). Two M1 strains isolated from humans with invasive infections have been described (Lannergård et al. 2011). All *S. pyogenes* strains were grown without shaking in Todd-Hewitt broth supplemented with 0.2% yeast extract (THY), in 5% CO_2_ at 37°C. Unless otherwise stated, the *S. pyogenes* cultures were supplemented with the cysteine protease inhibitor E64 (Sigma, St. Louis, Missouri, USA), used at 10 *μ*mol/L, to prevent degradation of surface-localized M protein by any SpeB protease released from the bacteria (Elliott 1945; Aziz et al. 2004; Wei et al. 2005; Gustafsson et al. 2013). Cloning work employed *Escherichia coli* XL1 and DH5*α*, whereas protein production employed strain BL21. Strains of *E. coli* were grown with shaking in LB at 37°C and supplemented with ampicillin (100 *μ*g mL^−1^) when appropriate.

### Proteins

Recombinant M proteins and M protein fragments were purified as GST-tagged proteins, as described (Gustafsson et al. 2013). After removal of the GST moiety, these proteins included the amino-terminal sequence GPLGS, not present in the original protein. Intact M1 and M5, and HVR and BCW fragments derived from these proteins, have been described (Lannergård et al. 2011; Gustafsson et al. 2013). For purification of the M1-(HVR-B), M1-CW, M5-B, and M5-CW fragments, the corresponding gene fragments were amplified as described (Gustafsson et al. 2013) from chromosomal DNA of strain SF370 or strain M5 Manfredo, with primers listed in Table S1, as follows: M1-(HVR-B): M1-F/M1B-dim-R2; M1-CW: M1C-F/M1-R; M5-B: M5B-F/M5B-dim-R; and M5-CW: M5C-F/M5-R. For isolation of the M1-(HVR-B-C) fragment, a supernatant of *S. pyogenes* strain MC25 was subjected to affinity chromatography on Fg immobilized in a HiTrap column (GE Healthcare) (Collin and Olsén 2000). Of note, the fragments M1-HVR, M1-(HVR-B), M5-HVR, and M5-B used here were dimerized by means of an added carboxy-terminal cysteine residue, not present in the native M protein, to enhance chances for coiled-coil formation and adoption of a native structure (Morfeldt et al. 2001; Sandin et al. 2002). The CW and BCW fragments were not dimerized by this procedure because the most carboxy-terminal part of an M protein does not form a coiled-coil (Nilson et al. 1995); however, the remaining part of these fragments is probably sufficient to promote coiled-coil formation (Gubbe et al. 1997). The M1 and M5 fragments include the following amino acid residues of the mature M proteins: M1-HVR: 1–91; M1-(HVR-B): 1–158; M1-(HVR-B-C): 1–330; M1-BCW: 92–443; M1-CW: 154–443; M5-HVR: 1–121; M5-B: 118–214; M5-BCW: 118–450; M5-CW: 209–450. Protein Rib was isolated from *Streptococcus agalactiae* strain BM110, as described (Stålhammar-Carlemalm et al. 1993; Larsson et al. 1996). Human Fg was from Enzyme Research Laboratories and protein G was from Sigma. Human elastase was from Elastin Products Company (Owensville, Missouri, USA) and streptococcal SpeB was from Toxin Technology (Sarasota, Florida, USA).

### Animal and human antisera to M proteins

#### Rabbit antisera

Antisera to proteins or peptides were raised by s.c. immunization with 100 *μ*g Ag in CFA, followed by two 50 *μ*g boosters in IFA. In the M1 system, anti-(M1-HVR) and anti-(M1-BCW) have been described (Lannergård et al. 2011). Anti-(M1-B) was raised by immunization with a synthetic 28-residue peptide, corresponding to the first B repeat and conjugated to the carrier OVA (Innovagen, Lund, Sweden). Anti-(M1-CW) was prepared by absorption of the anti-BCW serum on a HiTrap column containing the immobilized M1-(HVR-B) fragment. In the M5 system, two different antisera against the M5-HVR were used in different experiments, as indicated. One of these sera was raised against a recombinant fragment derived from the complete M5-HVR (Gustafsson et al. 2013); this antiserum was used for the cross-reactivity tests reported in Figure1B. The second anti-(M5-HVR) serum used here was raised against a synthetic peptide derived from residues 1–50 in the mature protein (Sandin et al. 2006); this antiserum was used in the tests shown in Figures 5 and S2. The anti-(M5-B) and anti-(M5-C) sera, raised against peptides derived from the B and C regions, respectively, have been described (Sandin et al. 2006).

#### Mouse sera

Antisera to pure M proteins were recovered from mice (C3H/HeN) that had been immunized with 20 *μ*g M1 or M5 mixed with alum, boosted with 10 *μ*g protein after 4 weeks, and bled 2 weeks later (Lannergård et al. 2011). Antisera from mice (C3H/HeN) infected with a sublethal dose of bacteria were recovered from animals infected i.p. 4 weeks earlier with 10^6^ cfu of strain M5 Manfredo (Lannergård et al. 2011) or with either of the M1 strains 5448 or 5448Δ*speB*.

#### Human sera

The two human convalescent sera studied here were recovered from patients who had been hospitalized for invasive *S. pyogenes* M1 infection (Lannergård et al. 2011). Of note, these patients were treated with a combination of clindamycin and benzylpenicillin at admission. As reported earlier, comparison of acute and convalescent sera demonstrated an increase in titer against M1, implying that the sera could be employed to analyze the human anti-M1 response during a single infection. Importantly, the corresponding M1 strains were available, allowing characterization of their M proteins.

### Binding and inhibition assays with antisera

The assays analyzed the ability of Abs, elicited by M1 or M5, to react with recombinant fragments derived from these proteins, and with the intact M proteins. In all binding tests, maximal binding was defined as 100%. Because the M1 protein has a weak IgG-Fc-binding activity (Åkesson et al. 1994), unlike M5, appropriate controls were included, as indicated, to ensure that this property did not affect the results. However, in our experience Fc-binding has limited effects in studies with purified M1. Of note, the HVR fragments of M1 and M5 used here had been dimerized by means of carboxy-terminal cysteine residue, unlike the HVR fragments employed previously (Lannergård et al. 2011), possibly explaining why the anti-HVR reactivity of some sera was slightly higher than in the analysis reported previously.

#### Analysis of cross-reactivity between M1 and M5

Microtiter wells were coated overnight at 4°C by incubation with a solution (50 *μ*L) of pure recombinant M1 or M5 (0.25 *μ*g mL^−1^ in PBS), as indicated. All subsequent steps were performed at room temperature (Fig.1B). After coating, the wells were blocked for 1 h with TBST-gel (Tris-buffered saline with 0.25% Tween-20 and 0.25% gelatin). Rabbit antiserum (50 *μ*L), diluted as indicated in TBST-gel, was then added and the wells were incubated for 1 h. After washes with PBSAT (PBS with 0.02% NaN_3_ and 0.05% Tween-20), radiolabeled protein G (∼10,000 cpm in 50 *μ*L TBST-gel) was added for detection of bound IgG, and after incubation for 1 h and new washes with PBSAT, the wells were cut out and bound protein G was determined in a *γ*-counter.

#### Direct binding of human and mouse Abs to M protein fragments

**Figure 2:**
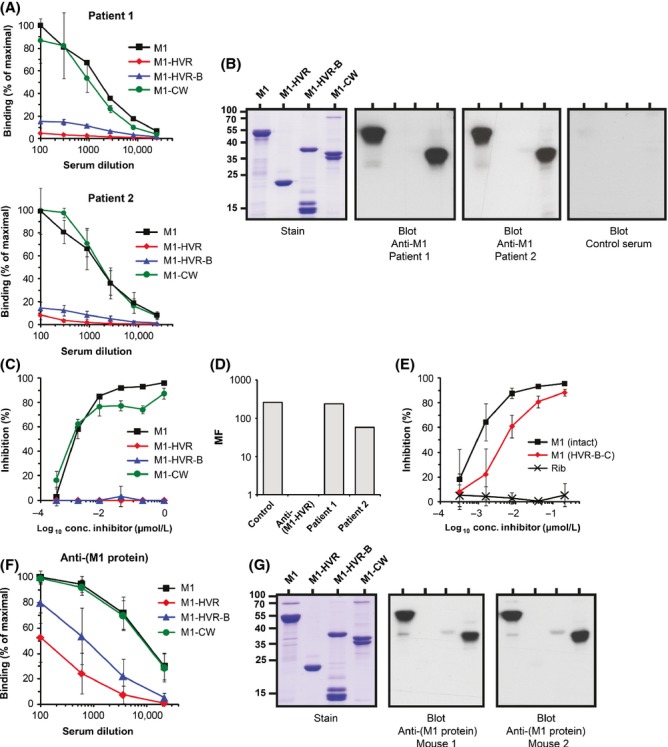
The entire HVR-B part of M1 is weakly immunogenic. (A) Analysis of convalescent sera from two patients, who were known to have responded to M1 during *Streptococcus pyogenes* infection. Each panel shows data obtained with one patient serum, as indicated. The human sera were tested for reactivity with the pure proteins indicated, immobilized in microtiter wells. Bound human Abs were detected with radiolabeled protein G. The assays had been optimized to allow direct comparisons between the curves. Of note, the Fc-binding ability of M1 was negligible under the conditions used here and did not influence the results. (B) Western blot analysis with the human convalescent sera and M1 fragments, as indicated. The analysis was performed under nonreducing conditions, because the HVR and HVR-B fragments had been dimerized via a carboxy-terminal cysteine residue, not present in the native M1 protein, to promote coiled-coil formation. This is reflected in the molecular masses observed. For unknown reasons, the dimerization of the HVR-B fragment was incomplete. Data are representative of two independent experiments. (C) Inhibition test with one of the human anti-M1 sera and M1 fragments. Reactivity of the human Abs with intact M1 immobilized in microtiter wells was inhibited with the fragments indicated. Similar data were obtained with the second human anti-M1 serum. (D) Opsonization-phagocytosis assay with whole human blood and the two human convalescent anti-M1 sera. Serum from a nonimmune human donor was used as a negative control and rabbit anti-(M1-HVR) was used to demonstrate that the system allowed opsonization. The figure shows average results based on two separate experiments, both of which yielded very similar results. MF, multiplication factor. (E) Inhibition test with intact M1 and its HVR-B-C fragment, which lacks most of the W region. The test system was the same as in panel C. An unrelated protein, streptococcal protein Rib, was used as negative control. The panel shows results obtained with one human serum; similar data were obtained with the second human serum. Controls with nonimmune human serum showed that Fc-binding was negligible in the tests shown in panels C and E. (F) Abs from mice (*n* = 9) immunized with pure M1 were tested for reactivity with proteins immobilized in microtiter wells, as indicated. Bound mouse Abs were detected by incubation with goat anti-(mouse Ig), and bound goat IgG was subsequently identified by incubation with radiolabeled protein G. No binding was observed with preimmune mouse serum, confirming that Fc-binding did not affect the results. (G) Western blot analysis with two of the mouse anti-M1 sera. Bound mouse Abs were detected as in F. Data are representative of two independent experiments. Data in panels A, C, E, and F show mean values ± SD and each analysis was performed three times.

**Figure 3:**
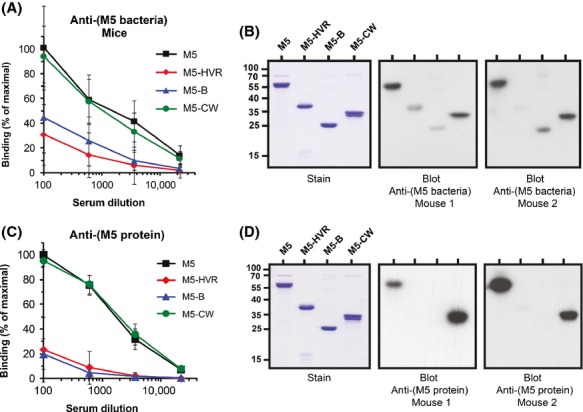
Also in the M5 protein the entire HVR-B part is weakly immunogenic. (A) Abs from mice (*n* = 8) infected with a sublethal dose of M5 bacteria were tested for reactivity with pure proteins immobilized in microtiter wells, as indicated. Bound mouse Abs were detected by incubation with rabbit anti-(mouse Ig), followed by protein G. Preimmune mouse serum lacked reactivity, reflecting the lack of Fc-reactivity of M5. (B) Western blot analysis with two of the sera from infected mice. Note that the HVR and B fragments had been dimerized via a carboxy-terminal cysteine residue, not present in the native M5 protein, to promote coiled-coil formation. The analysis was therefore performed under nonreducing conditions. The M5-HVR fragment migrated aberrantly, as previously observed for some dimerized HVRs (Morfeldt et al. 2001). Bound Abs were detected as in panel A. (C and D) Analysis of Abs from mice (*n* = 7) immunized with pure M5. The procedures used were similar to those in panels A and B. Data in panels A and C show mean values ± SD and each analysis was performed three times. The blotting data in panels B and D are representative of two independent experiments.

Microtiter wells were coated essentially as described above, using intact M protein and M protein fragments, as indicated (Fig.2A, F, 3A, C and Fig. S2). The protein concentrations used (0.3 to 3.5 *μ*g mL^−1^) had been optimized in preliminary experiments to give results allowing direct comparisons between data obtained for the different immobilized proteins (Lannergård et al. 2011). After blocking with TBST-gel, antisera were added at different dilutions, as indicated, followed by incubation for 1 h. Bound Abs were detected essentially as described above. For human sera, bound Abs were detected directly by incubation with radiolabeled protein G. For mouse sera, the wells were first incubated with goat anti-mouse Ig (Sigma, diluted 1000-fold) and then with protein G.

#### Inhibition assays with M protein fragments

**Figure 4:**
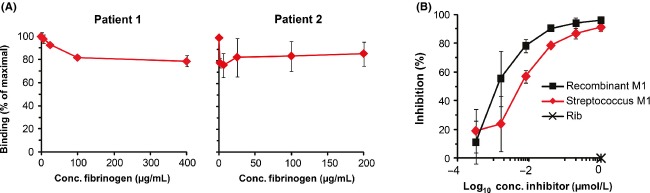
The weak antibody response does not reflect lack of antibody detection. (A) Search for possible ligand-induced binding sites (LIBS). Human convalescent anti-M1 serum was used to detect pure M1 immobilized in microtiter wells, and the effect of fibrinogen (Fg) on binding was determined. Data obtained with the two human sera are shown. (B) Search for possible modification affecting antigenicity. Human anti-M1 Abs, present in the serum of a convalescent patient, were used to detect immobilized M1, in this case the M1-(HVR-B-C) fragment isolated directly from *Streptococcus pyogenes*. Binding was inhibited with the same M1 preparation (“streptococcus M1”) and with recombinant M1 produced in *E. coli*. Streptococcal protein Rib, which is unrelated to M proteins, was used as negative control. Similar data were obtained with the second human convalescent serum. For panels A and B, controls with nonimmune human serum showed that Fc-binding did not affect the results. Data show mean values ± SD; each analysis was performed three times.

Microtiter wells were coated by incubation with a solution (50 *μ*L) of pure M1 (0.25–0.5 *μ*g mL^−1^) (Fig.2C, E and 4B). Human convalescent anti-M1 serum, diluted 300- or 600-fold, was preincubated for 30 min with the protein being tested for inhibitory ability, in a volume of 100 *μ*L. The samples were then analyzed for remaining Ab-binding activity, as described for direct binding tests. Of note, the Abs in a human control serum showed negligible binding under these conditions, demonstrating that the weak Fc-binding activity of M1 did not influence the result.

#### Effect of Fg on Ab-binding

Microtiter wells were coated overnight with a solution (50 *μ*L) of pure recombinant M1 (1.0 *μ*g mL^−1^), followed by the addition of Fg, as indicated, in a volume of 50 *μ*L and incubation for 1 h at room temperature (Fig.4A). Human convalescent anti-M1 serum, diluted 3000-fold, was then added in a volume of 50 *μ*L, and Ab-binding was determined, as described above. Serum from a nonimmune human donor showed low binding to M1 under these conditions, demonstrating that Fc-binding did not affect the results.

### Phagocytosis assays

The assay, based on the classical Todd-Lancefield test (Lancefield 1957), employed freshly drawn nonimmune human blood, that is blood allowing rapid growth of the strain used. Hirudin was used as anticoagulant (Thern et al. 1998). The assay employed a small inoculum (200–400 cfu) of log-phase bacteria, grown in THY without E64, and diluted in the same medium. The inoculum (25 *μ*L) was incubated for 10 min with 25 *μ*L test serum and 25 *μ*L of normal rabbit serum. Human blood (285 *μ*L) was then added. After rotation at 37°C for 3 h, each sample was plated on blood agar plates to determine bacterial concentration, and the multiplication factor (MF) was calculated. The normal rabbit serum was included because preliminary experiments indicated that it enhanced bacterial growth in the system. The positive control was a previously described rabbit anti-(M1-HVR) serum (Lannergård et al. 2011) and the negative control was serum from a human lacking opsonizing anti-M1 Abs. The two patient sera tested for opsonizing activity had been dialyzed against PBS, to remove any remaining antibiotics, and the two control sera were treated similarly.

### Protease digestions with bacteria

**Figure 5:**
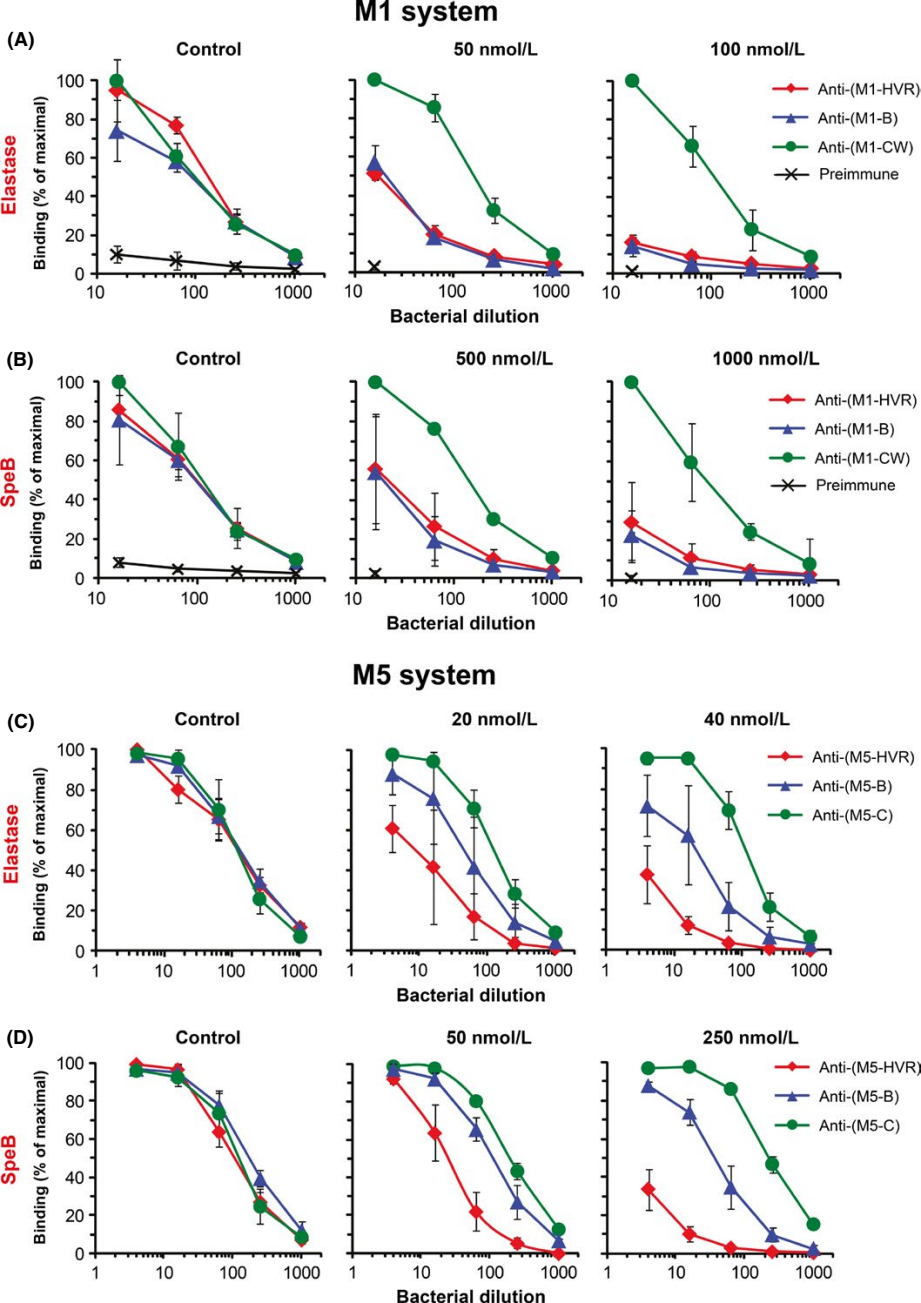
The variable part of bacteria-bound M1 or M5 is selectively eliminated by proteases. (A, B) M1-expressing *Streptococcus pyogenes* bacteria were either untreated (control) or treated with elastase or SpeB at the concentrations indicated. Different bacterial dilutions were subsequently tested for reactivity with a fixed amount of rabbit antiserum specific for the M1 region indicated. In this test, loss of an M protein region was seen as a reduction in binding of the corresponding rabbit Abs, resulting in a left-shift of the binding curve. To simplify comparisons, the three rabbit antisera had been adjusted to have similar reactivity with untreated bacteria (left panels). Binding of rabbit Abs was detected with radiolabeled protein G. As shown, preimmune rabbit serum showed little reactivity, demonstrating that IgG-Fc-binding to M1 was negligible in this test. (C, D) Treatment of M5-expressing *S. pyogenes* with elastase or SpeB. The analysis was performed as for panels A and B, employing rabbit antisera specific for the M5 regions indicated. Data show mean values ± SD; each analysis was performed three times.

The analysis employed strain SF370 (M1) or strain Manfredo (M5), as indicated, and an assay in which specific rabbit Abs were used to detect loss of an M protein region after protease digestion. Bacteria grown overnight in THY supplemented with E64 were washed 3 times in PBS and suspended to OD_620_ = 4. A sample (500 *μ*L) of the bacterial suspension was mixed with an equal volume of PBS containing elastase or SpeB, to obtain the final enzyme concentration indicated. For digests with SpeB, 10 mmol/L DTT was included, to promote activation of the enzyme. After incubation at 37°C for 1 h, the samples were chilled on ice and protease inhibitor was added to a final concentration of 1 mmol/L for the elastase-inhibitor AEBSF (Sigma) and 10 *μ*mol/L for the SpeB-inhibitor E64. All following steps were performed at room temperature. The bacteria were washed three times in PBSAT and serial dilutions were prepared, as indicated, in a suspension of *E. coli*, included to allow formation of a pellet after centrifugation. For analysis of remaining Ab-binding activity, a sample (90 *μ*L) of each bacterial dilution was mixed with a fixed amount (10 *μ*L) of the rabbit antiserum indicated, or with preimmune rabbit serum, used as control. The final dilution of rabbit antiserum used was based on preliminary optimization tests and was 300- to 1600-fold for the different sera. To simplify comparisons between results obtained with the three different antisera used, their concentrations were adjusted to yield similar results with undigested bacteria, as determined in preliminary tests (Fig.5, left panels). After incubation for 1 h with antiserum, the bacteria were washed twice with PBSAT, resuspended in PBSAT (100 *μ*L) containing ∼10,000 cpm radiolabeled protein G, and incubated for 1 h. The bacteria were then washed once with PBSAT and radioactivity in the pellets was determined in a *γ*-counter. Binding is presented as a percentage of the maximal binding obtained for each antiserum used. As the results are presented here, loss of an M protein region is reflected in a left-shift of the corresponding Ab-binding curve (Fig.5).

### Other methods

Radiolabeling of protein G with ^125^I, SDS-PAGE under reducing or nonreducing conditions, and Western blots were performed essentially as described (Stålhammar-Carlemalm et al. 1993; Lannergård et al. 2011). Protein sequence alignments were performed with the ClustalW program (http://www.ebi.ac.uk/Tools/msa/clustalw2/). For analysis of Fg-binding to M proteins and M protein fragments (Fig. S1), equimolar amounts of the bacterial proteins were used to coat microtiter wells, followed by incubation with Fg at the concentration indicated and detection of bound Fg with rabbit anti-Fg (Dako) and radiolabeled protein G.

### Ethics statement

Animal experiments were performed with permission from the Animal Experimental Ethics Committee at Lund District Court. Experimental infections were performed in a level P2 biohazard laboratory within the animal facility of the Department of Laboratory Medicine, Lund University. The serum samples from humans with invasive M1 infection were obtained with approval from the Research ethics committee at Karolinska Institutet and with written informed consent from the subjects or their legal guardians. Phagocytosis tests were performed with blood samples obtained from human volunteers, with permission from the Ethical Review Board of the Medical Faculty, Lund University and with written informed consent from the donors.

## Results

### Experimental system: the M1 and M5 proteins

Our work employed the intact M1 and M5 proteins and fragments derived from these proteins (Fig.1A). Comparative studies of these two clinically important M proteins were of interest because their HVRs and B repeat regions exhibit extreme sequence divergence and show little or no antigenic cross-reactivity (Fig.1A and B).

### The entire variable HVR-B part of M1 is weakly immunogenic in humans and mice

Previous analysis of the human Ab response to M1 indicated that most Abs were directed against a fragment designated BCW (comprising the B and C repeats and the W region), whereas the HVR elicited a weak response (Lannergård et al. 2011). This analysis was performed with sera obtained from two patients, for which both acute and convalescent serum and the infecting M1 strain were available, allowing the demonstration that the two patients had responded to the corresponding M1 protein during the recent infection. Further tests showed that the Abs in these human sera reacted equally well with intact M1 and its CW fragment but hardly at all with the HVR or HVR-B fragments, as judged by tests with proteins immobilized in microtiter wells (Fig.2A). Thus, the analysis indicated that not only the HVR, but the entire HVR-B part, was weakly immunogenic during human infection. Several lines of evidence supported this conclusion. First, the selective reactivity with intact M1 and the CW fragment could not be explained by the IgG-Fc-binding ability of M1 (Åkesson et al. 1994) because controls showed that <7% of the binding was caused by Fc-reactivity (data not shown). Second, only intact M1 and the CW fragment were detected in western blot analysis with the human convalescent sera, and no signal was obtained with a human control serum, confirming that the reactivity did not reflect IgG-Fc-binding (Fig.2B). Third, binding of the human Abs to M1 could be completely inhibited by intact M1 and the CW fragment, but not by the HVR or HVR-B fragment (Fig.2C). Fourth, the lack of reactivity with the HVR-B fragment did not reflect denaturation because this fragment and intact M1 had similar Fg-binding properties (Fig. S1A). Fifth, the lack of reactivity with the immobilized HVR and HVR-B fragments did not reflect a general inability to detect Abs to these fragments, as shown by controls [see (Lannergård et al. 2011) and Experimental Procedures]. Finally, the lack of detection of Abs to the HVR-B part did not reflect sequence variability in M1, as shown by analysis of DNA from the patient strains. This analysis showed that the patient strains and the reference strain expressed M1 proteins in which the HVR and B regions were identical, except for a single HVR residue that does not affect Ab recognition (Lannergård et al. 2011). Thus, a variety of analyses supported the conclusion that the human anti-M1 Abs were directed mainly against the conserved CW part. As discussed below, it is unlikely that the striking immunodominance of the conserved part can be explained as a recall response, reflecting previous *S. pyogenes* infection.

The HVR of an M protein is the major target for opsonizing Abs (Jones and Fischetti 1988; Fischetti 1989; Sandin et al. 2006), as determined in the whole blood assay commonly employed for *S. pyogenes* work (Lancefield 1957). The virtual absence of anti-HVR Abs in the two human sera therefore suggested that they would not promote phagocytosis in this assay. Both sera indeed lacked opsonizing capacity, while a hyperimmune rabbit anti-HVR serum promoted efficient killing, as expected (Fig.2D). Thus, both immunochemical and functional tests indicated that anti-HVR Abs were largely absent from the human M1 sera.

The W region of bacteria-bound M1 is probably hidden in the cell wall (Fischetti 1989), suggesting that anti-M1 Abs, elicited during an infection and detected with the CW fragment, might be directed mainly against the C repeat region. On the other hand, it seemed possible that some virtually intact M1 would be released from the bacteria, as described for protein A of *Staphylococcus aureus* (Becker et al. 2014), favoring the formation of Abs to the W region. However, binding of human anti-M1 Abs to M1 was inhibited not only by intact M1, but also by a truncated M1 protein lacking most of the W region (Fig.2E), indicating that the majority of the human anti-M1 Abs were directed against the C repeat region. For unknown reasons, the truncated M1 inhibited less efficiently than intact M1, but both proteins caused essentially complete inhibition, indicating that they contained the same epitopes.

The Ab response to pure M1 protein was analyzed in immunized mice. The Abs elicited in these mice reacted equally well with intact M1 and the CW fragment, but had low reactivity with the HVR and HVR-B fragments (Fig.2F), indicating that the entire variable part was weakly immunogenic also after immunization with pure protein. The binding to fragments derived from the variable part was somewhat higher for the mouse sera than for the human sera, but binding to the HVR was >50-fold lower and binding to the HVR-B fragment was at least 10-fold lower than to intact M1. No reactivity was seen in tests with preimmune mouse serum, confirming that the binding did not represent Fc-reactivity (not shown). Western blot analysis with two of the nine mouse sera supported the conclusion that the Ab response was largely directed against the conserved part of M1 (Fig.2G). Thus, the entire variable part of M1 was weakly immunogenic not only during human infection but also in immunized mice.

### The entire HVR-B part is weakly immunogenic also in the M5 protein

The Ab response to different regions in the M5 protein during infection was analyzed for mice infected with a sublethal dose of M5-expressing *S. pyogenes*. The anti-M5 Abs elicited in these infected mice reacted equally well with the CW fragment and intact M5 but had 15-40-fold lower reactivity with the HVR and the B fragment, as judged by tests with immobilized proteins (Fig.3A). The immunodominance of the conserved part was less dramatic in this system than in M1-infected humans (Fig.2A), but the overall picture was similar in the two systems. Thus, the analysis indicated that the entire variable HVR-B part of M5 is weakly immunogenic in infected mice. Western blot analysis with two of the eight mouse sera supported this conclusion (Fig.3B). Of note, the weak reactivity with the HVR and B fragments could not be explained by denaturation because the HVR retained ability to bind human factor H (Gustafsson et al. 2013), and the B fragment retained ability to bind Fg, although with reduced apparent affinity for Fg (Fig. S1B). As described for the M1 system, the weak response to the HVR and B regions did not reflect inability to detect Abs to these regions because the system had been optimized to allow detection of Abs against different M5 regions with the same sensitivity. Finally, the selective reactivity with intact M5 and the CW fragment did not reflect Fc-reactivity, because M5 lacks IgG-Fc-binding ability (Kehoe 1994).

The Ab response to pure M5 protein was analyzed in mice immunized with recombinant M5. The antisera reacted equally well with intact M5 and the CW fragment, but the reactivity with the HVR and the B fragment was ∼100-fold lower, according to tests with immobilized proteins (Fig.3C). In agreement with this result, western blot analysis with two of the seven mouse antisera showed lack of signal for the HVR and B fragments (Fig.3D). Thus, the entire variable HVR-B part of M5 was weakly immunogenic not only during infection but also after immunization with pure protein.

### Are antibodies to the variable part present but not detected?

An antigen that elicits a weak Ab response, as determined by standard methods, is by definition weakly immunogenic with regard to the humoral response. However, apparent lack of Abs could conceivably reflect lack of Ab detection rather than absence of Abs. In the M protein system studied here, this hypothesis would imply that the variable HVR-B part elicits Abs that largely go undetected in standard assays. We considered two hypothetical scenarios that could result in lack of Ab detection.

In one scenario, Abs to the variable part are directed mainly against epitopes that appear after the binding of a host ligand, resulting in apparent lack of Abs when analysis is performed in the absence of the ligand. Studies with certain monoclonal Abs have allowed the identification of such ligand-induced binding sites (LIBS) in ligand-receptor systems (Frelinger et al. 1990; Speziale et al. 1996), but we are not aware of any system where lack of ligand-binding can explain why a polyclonal Ab response appears to be weak. This problem was nevertheless studied in the M1 system, focusing on the Fg-binding B repeat region. Specifically, we analyzed whether human anti-M1 sera showed increased reactivity with pure M1 when Fg was added. In this analysis, Fg did not stimulate binding but caused a slight inhibition of binding for each of the two patient sera (Fig.4A). Similar results were obtained in the M5 system, with sera obtained from mice immunized with pure M5 (data not shown). Thus, analysis in the M1 and M5 systems did not support the hypothesis that Abs to the variable part went undetected because the analysis was performed in the absence of ligand.

In another possible scenario, Abs to the variable part were not detected, because this part is only immunogenic after having been structurally modified, which might occur in streptococci. Accordingly, Abs against the modified variable part would not have been detected, because recombinant unmodified protein was used in immunochemical tests. Moreover, the hypothesis predicts that the unmodified variable part present in recombinant protein would not elicit Abs. Such a remarkable situation was recently described for a group of *Staphylococcus aureus* proteins (Hazenbos et al. 2013). To analyze whether M protein has similar properties, we employed the human Abs described above to detect immobilized M1 protein isolated directly from *S. pyogenes*. If a considerable fraction of the human Abs detect a modified form of M1, it should be possible to efficiently inhibit this binding with M1 protein isolated directly from streptococci, but not with recombinant M1. However, M1 preparations of both types caused virtually complete inhibition (Fig.4B). Thus, the analysis did not provide support for the hypothesis that lack of Ab detection reflects structural modification.

### The variable part of bacteria-bound M protein is selectively eliminated by proteases

We hypothesized that the weak immunogenicity of the variable part in M1 and M5 resulted from sensitivity to proteases. In this scenario, the variable part plays a key role in pathogenesis but is also rapidly eliminated by protease attack, resulting in a limited Ab response and immune escape. To analyze this hypothesis, we employed human elastase, the potent serine protease of neutrophils (Pham 2006), and SpeB, the well-known cysteine protease of *S. pyogenes* (Nelson et al. 2011). Elastase was studied because it is a major protease in inflamed tissue, whereas SpeB was studied because it is secreted from *S. pyogenes* and may attack surface proteins on the same bacteria, in particular M protein (Elliott 1945; Berge and Björck 1995; Wei et al. 2005). Attack by SpeB cannot affect the Ab response when pure M protein is used for immunization, but this enzyme was nevertheless studied, because it could affect the response during an infection, when large amounts of SpeB might be present in the bacterial microenvironment.

Our analysis was initially focused on pure M proteins. In such analysis, incubation of pure M proteins with proteases at 37° did not allow demonstration of selective sensitivity of the variable part. This result was not unexpected because the two-coiled coil chains of pure M protein are known to dissociate at 37°, probably resulting in loss of secondary and tertiary structure (Åkerström et al. 1992; Cedervall et al. 1995; Nilson et al. 1995). In an attempt to circumvent this problem, incubations were performed at 25°, but again with inconclusive results, possibly due to the unphysiological conditions used. These results suggested that analysis should be performed under conditions more similar to those encountered in vivo and focused interest on the ability of bound plasma proteins to stabilize the dimeric form of M proteins at 37° (Cedervall et al. 1995; Nilson et al. 1995). However, protease digestion in the presence of plasma was not attempted, because an Fg-binding M protein will form precipitates with the Fg present in plasma (Kantor 1965). This situation prompted us to perform further work with whole bacteria, in which covalent binding of M protein to the cell wall (Schneewind and Missiakas 2012) likely limits chain dissociation (Cedervall et al. 1995).

Bacteria were cultivated in broth supplemented with the cysteine protease inhibitor E64, to avoid possible degradation of M protein by SpeB secreted into the culture (Elliott 1945; Aziz et al. 2004; Wei et al. 2005; Gustafsson et al. 2013). Washed bacteria were subsequently treated with pure elastase or SpeB, and loss of an M protein region was determined as loss of reactivity with rabbit Abs against the corresponding region (Fig.5). Of note, the rabbit Abs used for this analysis had been shown to be specific for the corresponding region of the M protein studied (Fig. S2). As presented here, loss of an M protein region after protease treatment is reflected in a left-shift of the corresponding Ab-binding curve. For each enzyme, we employed two different concentrations, chosen on the basis of preliminary experiments.

For M1-expressing bacteria, the different rabbit antisera had been adjusted to have the same reactivity with untreated control bacteria, to simplify comparisons (Fig.5A and B, left panels). After enzyme treatment, a different picture emerged. In tests with elastase, treatment with 50 nmol/L enzyme caused a strong loss in reactivity with anti-HVR and anti-B (Fig.5A, middle panel), and treatment with 100 nmol/L enzyme caused almost complete loss of reactivity with these two antisera (Fig.5A, right panel). In contrast, reactivity with anti-CW was virtually unchanged after enzyme treatment. Of note, preimmune rabbit serum had negligible binding activity in this assay, demonstrating that the weak Fc-binding ability of M1 did not influence the results. Similar results were obtained with SpeB tested at two concentrations (Fig.5B). Thus, the entire variable HVR-B part of bacteria-bound M1 was selectively eliminated by treatment with elastase or SpeB, whereas the conserved part remained largely unaffected.

The data on SpeB suggested that attack by this protease might affect the Ab response to M1 during an infection. To analyze this hypothesis, we infected two groups of mice with sublethal doses of the M1 strain 5448 and an SpeB-negative mutant of that strain, respectively. Only some of the infected mice responded to M1, but analysis of two sera from each group indicated that the Ab response to the variable part was similar for all mice analyzed, that is it was weak also in the absence of SpeB (Fig. S3). Thus, attack by SpeB alone is not sufficient to explain the weak Ab response to the variable part of M1 during experimental infection.

In the M5 system, the effects of proteases on bacteria-bound protein were similar but not identical to those obtained in the M1 system. In tests with elastase, the HVR was largely lost by enzyme treatment, whereas the conserved part was unaffected but the B region showed intermediate sensitivity (Fig.5C). Similar results were obtained with SpeB (Fig.5D). Thus, a gradient of protease sensitivity could be discerned in the M5 system, but the overall picture was very similar to that observed in the M1 system.

## Discussion

The data described here and in an earlier report (Lannergård et al. 2011) challenge the common assumption that a highly variable region in a microbial virulence factor elicits a stronger Ab response than other parts of the protein. On the contrary, our data support the paradoxical hypothesis that some variable regions are weakly immunogenic, because they are so important for virulence, that evolution has favored the appearance of at least two mechanisms that limit Ab attack on these regions, sequence variability and weak immunogenicity.

Our work employed the streptococcal M1 and M5 proteins, which have similar overall structure, with HVR, B, C, and W regions. In previous studies of these M proteins, we compared the Ab responses to the HVR and the remaining part of the M protein (designated BCW) and found that that the HVR elicited a weak Ab response, whereas the BCW part was immunodominant (Lannergård et al. 2011). Thus, our data supported the hypothesis that a highly variable region may escape Abs by being weakly immunogenic. However, the situation has remained unclear for the B repeat region. The extensive sequence divergence in this Fg-binding region suggested to us that it is subject to immune pressure and might have the same immunological properties as the HVR. In agreement with this hypothesis, we found that the B repeats of M1 and M5 indeed elicited a weak Ab response. In contrast, it has been proposed that the B repeat region is immunodominant in the M6 protein (Fischetti and Windels 1988), for which the B repeats are virtually identical to those in M5 (Miller et al. 1988). However, the conclusions of the M6 study were mainly based on work with short synthetic peptides, which may have aberrant immunological properties, and tests with larger fragments reported in that study do not disagree with the conclusion that the B region is weakly immunogenic also in M6. A coherent picture is therefore emerging, indicating that the entire variable part of an M protein elicits a weak Ab response.

The antisera studied here were obtained from patients and from infected mice, and from mice immunized with pure M1 or M5. In all cases, the results supported the conclusion that not only the HVR but also the B repeat region was weakly immunogenic, whereas the conserved part was immunodominant. For the human sera, it is possible that a recall response contributed to the result, reflecting previous *S. pyogenes* infection(s), but a recall response cannot explain the results obtained with mice. Moreover, there was virtually no response to the variable HVR-B part of M1 in the convalescent sera studied here, favoring the notion that this part is weakly immunogenic in humans. The inability of the human sera to promote phagocytosis supports the conclusion that they had low levels of anti-HVR Abs. This finding agrees well with early reports that type-specific opsonizing Abs appear only after prolonged *S. pyogenes* infection (Denny et al. 1957; Lancefield 1959) and are not found in patients treated with penicillin (Daikos and Weinstein 1951; Denny et al. 1957; Siegel et al. 1961). Importantly, the weak Ab response to the variable part of an M protein may limit the development of herd immunity, possibly allowing reinfection with strains of the same M type.

Several explanations may be envisaged for the weak immunogenicity of the variable part of an M protein, including lack of defined structure (Dey et al. 2009), selective elimination by proteases, and limited affinity maturation of the corresponding B cells (Pauli et al. 2014). Among these alternatives, our interest was focused on protease sensitivity, a property traditionally attributed to the type-specific part of an M protein (Lancefield 1943). It was not technically possible to perform studies with pure M proteins, but in tests with intact bacteria we found that the variable part of M protein indeed was selectively eliminated by digestion with elastase or SpeB, while the conserved part was resistant. This finding supports the hypothesis that weak immunogenicity is a result of protease attack and provides a possible explanation for the surprising fact that M protein is sensitive to proteases, unlike many other bacterial surface proteins. Indeed, protease sensitivity of the variable part may be an essential feature of M protein, allowing it to evade host immunity.

Host proteases that might attack M protein during *S. pyogenes* infection include elastase and other neutrophil proteases, and plasminogen activated by bacterial streptokinase (Sun et al. 2004). Our studies indicated that attack by streptococcal SpeB is not sufficient to explain the weak immunogenicity of the variable part during infection, but this result does not exclude that SpeB is one of several proteases that attack M protein in this setting. Molecular features that might favor attack on the variable part include local irregularities in the coiled-coil (Nilson et al. 1995; McNamara et al. 2008), the wall-distal location of the variable part, and protection of the conserved part by bound albumin (Åkesson et al. 1994; Retnoningrum and Cleary 1994; Sandin et al. 2006). The fate of the variable region after protease attack is not known, but a well-defined fragment, similar to the pepM fragment released after treatment of *S. pyogenes* with pepsin under suboptimal conditions (Beachey et al. 1977), was not identified in our digests (data not shown). This result does not exclude that M protein fragments might be liberated in vivo and could have important biologic effects, even if they are not immunogenic (Herwald et al. 2004; Schmitt et al. 2010).

Formal proof that the weak immunogenicity of the variable part reflects protease sensitivity may require the demonstration that a mutant M protein, which lacks protease sensitive sites, elicits a good Ab response to the variable region. It remains uncertain whether such a mutant protein can be constructed and preliminary attempts to identify a protease sensitive site were unsuccessful. Nevertheless, our findings demonstrate an intriguing correlation between weak immunogenicity and proteolytic elimination, supporting the notion that these properties are functionally related.

Any mechanism that allows *S. pyogenes* to evade Ab attack has most likely evolved at mucosal sites and other epithelial surfaces, where immune escape provides a selective advantage by enhancing chances for transmission to a new host (Anderson and May 1986). To explain the data reported here, we therefore propose that the variable HVR and B regions of M1 and M5 have key functions during the establishment of infections at epithelial surfaces and are subjected to immune pressure in that setting, but also are rapidly eliminated through proteolytic attack and therefore are weakly immunogenic. Accordingly, it would be of particular interest to analyze the Ab response to M protein in mucosal and other epithelial microenvironments, but such analysis is not yet feasible. Our study was therefore based on studies of serum Abs, which may be used to evaluate local immunity, because several lines of evidence indicate that induction of mucosal immunity results in serum IgG responses (Lee et al. 2003; Takahashi et al. 2009). Similarly, serum IgG induced by parenteral vaccination confers protection at mucosal surfaces, a prerequisite for the success of many vaccines (Robbins et al. 1995; Brandtzaeg 2007). Thus, the intimate connection between mucosal and systemic immunity allows studies of serum Abs to be used as a surrogate for the analysis of locally produced Abs. Of note, studies of serum Abs are also highly relevant for understanding of the response to vaccinations.

The hypothesis, that the HVR and B regions play key roles at epithelial surfaces, does not exclude that they also promote virulence during invasive infection (Waldemarsson et al. 2009) but focuses interest on the possible role of these regions at epithelia. The function of the HVRs in M1 and M5 remains unclear, but they might promote adhesion to epithelial cells (Wang and Stinson 1994; Penfound et al. 2010), a key step in pathogenesis that should be highly vulnerable to Ab attack. If the HVR promotes adhesion, an additional role for protease attack could be to promote bacterial dispersal in the infected tissue (Connolly et al. 2011). In the M5 system, the ability of the HVR to bind human factor H may contribute to virulence, but the role of factor H in *S. pyogenes* infection remains unclear (Gustafsson et al. 2013). For the B repeat region, the main function is probably to inhibit phagocytosis (Carlsson et al. 2005), a function that may be of major importance during the establishment of an infection at an epithelial surface, although phagocytosis resistance commonly is analyzed in a whole blood assay (Lancefield 1957). Accordingly, the B repeat region could be an important target for protective Abs at epithelia. In blood, Fg apparently competes with Abs for binding to the B repeats (Sandin et al. 2006), but the low concentration of Fg in secretions such as saliva should limit the opportunity for competition at epithelial surfaces (Berckmans et al. 2011).

In summary, our studies of the M1 and M5 proteins indicate that each of the highly variable HVR and B repeat regions elicits a weak Ab response, a property that might be explained by protease attack. These findings support the notion that the entire variable HVR-B part evades Abs not only by sequence variability but also by weak immunogenicity, and they focus interest on weak immunogenicity as an immune escape mechanism that potentially is of general importance.

## Supporting Information

**Figure S1.** Binding of fibrinogen to the HVR-B fragment of M1 and the B fragment of M5. **Figure S2.** Specificity of rabbit antisera used to detect different regions in M1 and M5. **Figure S3.** Absence of SpeB does not affect the Ab response to M1 during experimental infection. **Table S1.** Primers used for PCR amplification.

## References

[b1] Åkerström B, Lindahl G, Björck L, Lindqvist A (1992). Protein Arp and protein H from group A streptococci. Ig binding and dimerization are regulated by temperature. J. Immunol.

[b2] Åkesson P, Schmidt KH, Cooney J, Björck L (1994). M1 protein and protein H: igGFc- and albumin-binding streptococcal surface proteins encoded by adjacent genes. Biochem. J.

[b3] Anderson RM, May RM (1986). The invasion, persistence and spread of infectious diseases within animal and plant communities. Philos. Trans. R. Soc. Lond. B Biol. Sci.

[b4] Aziz RK, Pabst MJ, Jeng A, Kansal R, Low DE, Nizet V (2004). Invasive M1T1 group A Streptococcus undergoes a phase-shift in vivo to prevent proteolytic degradation of multiple virulence factors by SpeB. Mol. Microbiol.

[b5] Beachey EH, Stollerman GH, Chiang EY, Chiang TM, Seyer JM, Kang AH (1977). Purification and properties of M protein extracted from group A streptococci with pepsin: covalent structure of the amino terminal region of type 24 M antigen. J. Exp. Med.

[b6] Becker S, Frankel MB, Schneewind O, Missiakas D (2014). Release of protein A from the cell wall of *Staphylococcus aureus*. Proc. Natl Acad. Sci. USA.

[b7] Berckmans RJ, Sturk A, van Tienen LM, Schaap MC, Nieuwland R (2011). Cell-derived vesicles exposing coagulant tissue factor in saliva. Blood.

[b8] Berge A, Björck L (1995). Streptococcal cysteine proteinase releases biologically active fragments of streptococcal surface proteins. J. Biol. Chem.

[b9] Brandtzaeg P (2007). Do salivary antibodies reliably reflect both mucosal and systemic immunity?. Ann. N. Y. Acad. Sci.

[b10] Carapetis JR, Steer AC, Mulholland EK, Weber M (2005). The global burden of group A streptococcal diseases. Lancet Infect. Dis.

[b11] Carlsson F, Sandin C, Lindahl G (2005). Human fibrinogen bound to *Streptococcus pyogenes* M protein inhibits complement deposition via the classical pathway. Mol. Microbiol.

[b12] Cedervall T, Åkesson P, Stenberg L, Herrmann A, Åkerström B (1995). Allosteric and temperature effects on the plasma protein binding by streptococcal M protein family members. Scand. J. Immunol.

[b13] Collin M, Olsén A (2000). Generation of a mature streptococcal cysteine proteinase is dependent on cell wall-anchored M1 protein. Mol. Microbiol.

[b14] Connolly KL, Roberts AL, Holder RC, Reid SD (2011). Dispersal of group A streptococcal biofilms by the cysteine protease SpeB leads to increased disease severity in a murine model. PLoS ONE.

[b15] Daikos G, Weinstein L (1951). Streptococcal bacteriostatic antibody in patients treated with penicillin. Proc. Soc. Exp. Biol. Med.

[b16] Dale JB, Penfound TA, Chiang EY, Walton WJ (2011). New 30-valent M protein-based vaccine evokes cross-opsonic antibodies against non-vaccine serotypes of group A streptococci. Vaccine.

[b17] Denny FW, Perry WD, Wannamaker LW (1957). Type-specific streptococcal antibody. J. Clin. Invest.

[b18] Dey B, Svehla K, Xu L, Wycuff D, Zhou T, Voss G (2009). Structure-based stabilization of HIV-1 gp120 enhances humoral immune responses to the induced co-receptor binding site. PLoS Pathog.

[b19] Elliott SD (1945). A proteolytic enzyme produced by group A streptococci with special reference to its effect on the type-specific M antigen. J. Exp. Med.

[b20] Ferretti JJ, McShan WM, Ajdic D, Savic DJ, Savic G, Lyon K (2001). Complete genome sequence of an M1 strain of *Streptococcus pyogenes*. Proc. Natl Acad. Sci. USA.

[b21] Fischetti VA (1989). Streptococcal M protein: molecular design and biological behavior. Clin. Microbiol. Rev.

[b22] Fischetti VA, Windels M (1988). Mapping the immunodeterminants of the complete streptococcal M6 protein molecule. Identification of an immunodominant region. J. Immunol.

[b23] Frelinger AL, Cohen I, Plow EF, Smith MA, Roberts J, Lam SC (1990). Selective inhibition of integrin function by antibodies specific for ligand-occupied receptor conformers. J. Biol. Chem.

[b24] Gubbe K, Misselwitz R, Welfle K, Reichardt W, Schmidt KH, Welfle H (1997). C repeats of the streptococcal M1 protein achieve the human serum albumin binding ability by flanking regions which stabilize the coiled-coil conformation. Biochemistry.

[b25] Gustafsson MC, Lannergård J, Nilsson OR, Kristensen BM, Olsen JE, Harris CL (2013). Factor H binds to the hypervariable region of many *Streptococcus pyogenes* M proteins but does not promote phagocytosis resistance or acute virulence. PLoS Pathog.

[b26] Hazenbos WL, Kajihara KK, Vandlen R, Morisaki JH, Lehar SM, Kwakkenbos MJ (2013). Novel staphylococcal glycosyltransferases SdgA and SdgB mediate immunogenicity and protection of virulence-associated cell wall proteins. PLoS Pathog.

[b27] Herwald H, Cramer H, Mörgelin M, Russell W, Sollenberg U, Norrby-Teglund A (2004). M protein, a classical bacterial virulence determinant, forms complexes with fibrinogen that induce vascular leakage. Cell.

[b28] Jones KF, Fischetti VA (1988). The importance of the location of antibody binding on the M6 protein for opsonization and phagocytosis of group A M6 streptococci. J. Exp. Med.

[b29] Kansal RG, Nizet V, Jeng A, Chuang WJ, Kotb M (2003). Selective modulation of superantigen-induced responses by streptococcal cysteine protease. J. Infect. Dis.

[b30] Kantor FS (1965). Fibrinogen precipitation by streptococcal M protein. I. Identity of the reactants, and stoichiometry of the reaction. J. Exp. Med.

[b31] Kehoe MA (1994). Cell-wall-associated proteins in Gram-positive bacteria. New Comp. Biochem.

[b32] Lancefield RC (1943). Studies on the antigenic composition of group A hemolytic streptococci: I. Effects of proteolytic enzymes on streptococcal cells. J. Exp. Med.

[b33] Lancefield RC (1957). Differentiation of group A streptococci with a common R antigen into three serological types, with special reference to the bactericidal test. J. Exp. Med.

[b34] Lancefield RC (1959). Persistence of type-specific antibodies in man following infection with group A streptococci. J. Exp. Med.

[b35] Lannergård J, Gustafsson MC, Waldemarsson J, Norrby-Teglund A, Stålhammar-Carlemalm M, Lindahl G (2011). The hypervariable region of *Streptococcus pyogenes* M protein escapes antibody attack by antigenic variation and weak immunogenicity. Cell Host Microbe.

[b36] Larsson C, Stålhammar-Carlemalm M, Lindahl G (1996). Experimental vaccination against group B streptococcus, an encapsulated bacterium, with highly purified preparations of cell surface proteins Rib and alpha. Infect. Immun.

[b37] Lee CJ, Lee LH, Frasch CE (2003). Protective immunity of pneumococcal glycoconjugates. Crit. Rev. Microbiol.

[b38] McMillan DJ, Dreze PA, Vu T, Bessen DE, Guglielmini J, Steer AC (2013). Updated model of group A Streptococcus M proteins based on a comprehensive worldwide study. Clin. Microbiol. Infect.

[b39] McNamara C, Zinkernagel AS, Macheboeuf P, Cunningham MW, Nizet V, Ghosh P (2008). Coiled-coil irregularities and instabilities in group A Streptococcus M1 are required for virulence. Science.

[b40] Miller L, Gray L, Beachey E, Kehoe M (1988). Antigenic variation among group A streptococcal M proteins. Nucleotide sequence of the serotype 5 M protein gene and its relationship with genes encoding types 6 and 24 M proteins. J. Biol. Chem.

[b41] Morfeldt E, Berggård K, Persson J, Drakenberg T, Johnsson E, Lindahl E (2001). Isolated hypervariable regions derived from streptococcal M proteins specifically bind human C4b-binding protein: implications for antigenic variation. J. Immunol.

[b42] Nelson DC, Garbe J, Collin M (2011). Cysteine proteinase SpeB from *Streptococcus pyogenes* - a potent modifier of immunologically important host and bacterial proteins. Biol. Chem.

[b43] Nilson BH, Frick IM, Åkesson P, Forsén S, Björck L, Åkerström B (1995). Structure and stability of protein H and the M1 protein from *Streptococcus pyogenes*. Implications for other surface proteins of gram-positive bacteria. Biochemistry.

[b44] Pauli NT, Kim HK, Falugi F, Huang M, Dulac J, Henry Dunand C (2014). *Staphylococcus aureus* infection induces protein A-mediated immune evasion in humans. J. Exp. Med.

[b45] Penfound TA, Ofek I, Courtney HS, Hasty DL, Dale JB (2010). The NH(2)-terminal region of *Streptococcus pyogenes* M5 protein confers protection against degradation by proteases and enhances mucosal colonization of mice. J. Infect. Dis.

[b46] Persson J, Beall B, Linse S, Lindahl G (2006). Extreme sequence divergence but conserved ligand-binding specificity in *Streptococcus pyogenes* M protein. PLoS Pathog.

[b47] Pham CT (2006). Neutrophil serine proteases: specific regulators of inflammation. Nat. Rev. Immunol.

[b48] Retnoningrum DS, Cleary PP (1994). M12 protein from *Streptococcus pyogenes* is a receptor for immunoglobulin G3 and human albumin. Infect. Immun.

[b49] Robbins JB, Schneerson R, Szu SC (1995). Perspective: hypothesis: serum IgG antibody is sufficient to confer protection against infectious diseases by inactivating the inoculum. J. Infect. Dis.

[b50] Sanderson-Smith M, De Oliveira DM, Guglielmini J, McMillan DJ, Vu T, Holien JK (2014). A systematic and functional classification of *Streptococcus pyogenes* that serves as a new tool for molecular typing and vaccine development. J. Infect. Dis.

[b51] Sandin C, Linse S, Areschoug T, Woof JM, Reinholdt J, Lindahl G (2002). Isolation and detection of human IgA using a streptococcal IgA-binding peptide. J. Immunol.

[b52] Sandin C, Carlsson F, Lindahl G (2006). Binding of human plasma proteins to *Streptococcus pyogenes* M protein determines the location of opsonic and non-opsonic epitopes. Mol. Microbiol.

[b53] Schmitt R, Carlsson F, Mörgelin M, Tati R, Lindahl G, Karpman D (2010). Tissue deposits of IgA-binding streptococcal M proteins in IgA nephropathy and Henoch-Schonlein purpura. Am. J. Pathol.

[b54] Schneewind O, Missiakas DM (2012). Protein secretion and surface display in Gram-positive bacteria. Philos. Trans. R. Soc. Lond. B Biol. Sci.

[b55] Siegel AC, Johnson EE, Stollerman GH (1961). Controlled studies of streptococcal pharyngitis in a pediatric population. 2. Behavior of the type-specific immune response. N. Engl. J. Med.

[b56] Speziale P, Joh D, Visai L, Bozzini S, House-Pompeo K, Lindberg M (1996). A monoclonal antibody enhances ligand binding of fibronectin MSCRAMM (adhesin) from *Streptococcus dysgalactiae*. J. Biol. Chem.

[b57] Stålhammar-Carlemalm M, Stenberg L, Lindahl G (1993). Protein Rib: a novel group B streptococcal cell surface protein that confers protective immunity and is expressed by most strains causing invasive infections. J. Exp. Med.

[b58] Steer AC, Law I, Matatolu L, Beall BW, Carapetis JR (2009). Global *emm* type distribution of group A streptococci: systematic review and implications for vaccine development. Lancet Infect. Dis.

[b59] Sun H, Ringdahl U, Homeister JW, Fay WP, Engleberg NC, Yang AY (2004). Plasminogen is a critical host pathogenicity factor for group A streptococcal infection. Science.

[b60] Takahashi I, Nochi T, Yuki Y, Kiyono H (2009). New horizon of mucosal immunity and vaccines. Curr. Opin. Immunol.

[b61] Thern A, Wästfelt M, Lindahl G (1998). Expression of two different antiphagocytic M proteins by *Streptococcus pyogenes* of the OF+ lineage. J. Immunol.

[b62] Waldemarsson J, Stålhammar-Carlemalm M, Sandin C, Castellino FJ, Lindahl G (2009). Functional dissection of *Streptococcus pyogenes* M5 protein: the hypervariable region is essential for virulence. PLoS ONE.

[b63] Wang JR, Stinson MW (1994). M protein mediates streptococcal adhesion to HEp-2 cells. Infect. Immun.

[b64] Wei L, Pandiripally V, Gregory E, Clymer M, Cue D (2005). Impact of the SpeB protease on binding of the complement regulatory proteins factor H and factor H-like protein 1 by *Streptococcus pyogenes*. Infect. Immun.

