# Supplementary material for: Sequence variability is correlated with weak immunogenicity in Streptococcus pyogenes M protein
Source: Microbiologyopen. 2015 Jul 15;4(5):774–89. doi: 10.1002/mbo3.278 (PMC4618610; doi:10.1002/mbo3.278)
Supplement: Supplementary file 1 — Figure S1. Binding of fibrinogen to the HVR-B fragment of M1 and the B fragment of M5. Figure S2. Specificity of rabbit antisera used to detect different regions in M1 and M5. Figure S3. Absence of SpeB does not affect the Ab response to M1 during experimental infection. Table S1. Primers used for PCR amplification. [file mbo30004-0774-sd1.pdf]

SUPPORTING INFORMATION for

Sequence variability is correlated to weak immunogenicity in *Streptococcus pyogenes* M protein

by

Jonas Lannergård, Bodil M. Kristensen, Mattias C. U. Gustafsson, Jenny J. Persson, Anna Norrby-Teglund, Margaretha Stålhammar-Carlemalm, and Gunnar Lindahl

Fig. S1. Binding of fibrinogen to the HVR-B fragment of M1 and the B fragment of M5.

Fig. S2. Specificity of rabbit antisera used to detect different regions in M1 and M5.

Fig. S3. Absence of SpeB does not affect the Ab response to M1 during experimental infection.

Table S1. Primers used for PCR amplification.

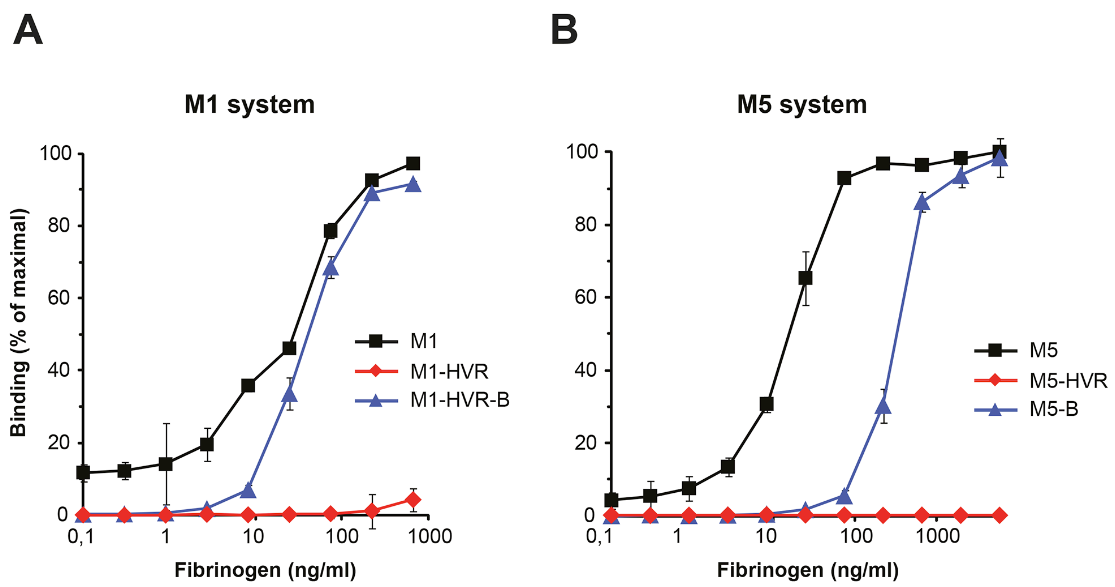

Fig. S1. Binding of fibrinogen to the HVR-B fragment of M1 and the B fragment of M5.

A. Microtiter wells were coated with equimolar amounts of the M1 protein and its two fragments HVR and HVR-B. The ability of these proteins to bind fibrinogen (Fg) was evaluated through the addition of Fg at increasing concentration, as indicated. Bound Fg was identified by incubation with rabbit anti-Fg followed by radiolabeled protein G. The HVR-B fragment, but not the HVR, had Fg-binding properties similar to those of intact M1. The apparent binding to M1 at very low concentrations of Fg represents Fc-binding of rabbit Abs to M1.

B. Similar analysis in the M5 system. Intact M5 and its B fragment, but not the HVR, showed binding of Fg, but binding was weaker for the B fragment. Data show mean values ± SD; each analysis was performed three times.

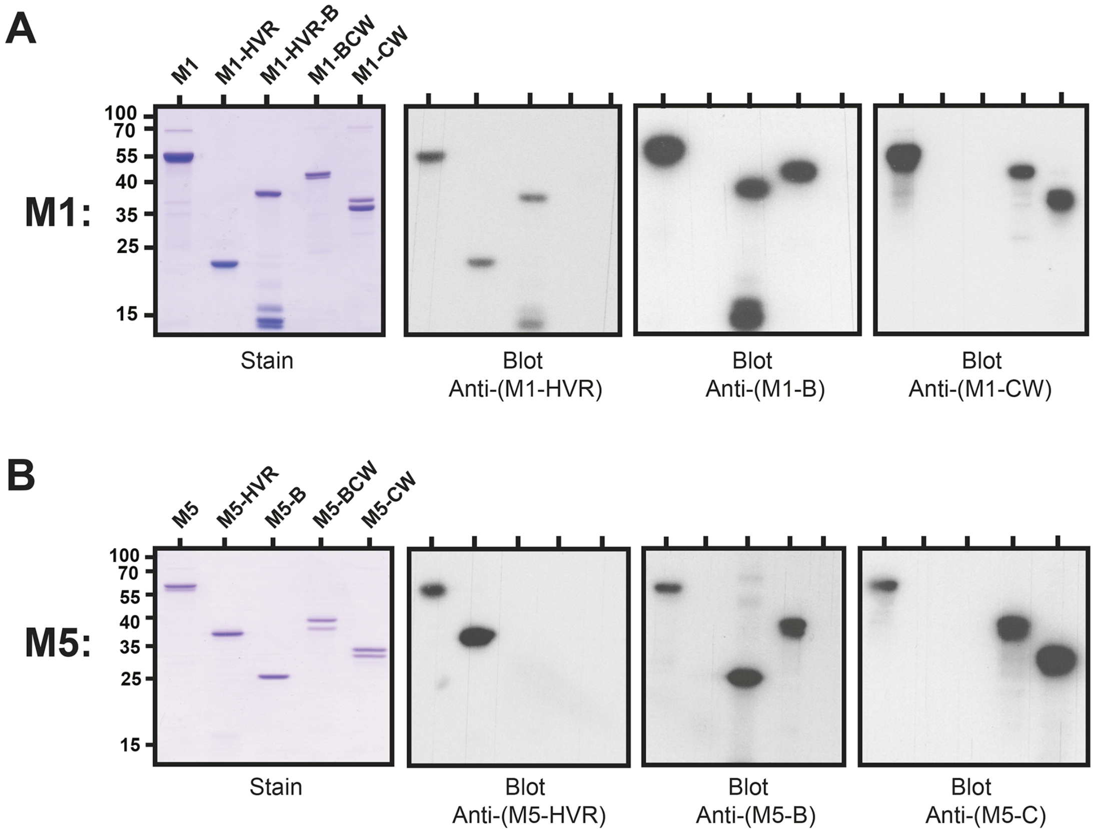

Fig. S2. Specificity of rabbit antisera used to detect different regions in M1 and M5.

A. Intact M1 and a series of M1 fragments were analyzed for reactivity with the rabbit antisera indicated, raised against M protein-derived polypeptides. The M1 fragments used are described in Figure 1, except for the previously described BCW fragment, which comprises the B, C and W regions of M1. The analysis was performed by western blot, using radiolabeled protein G to detect bound rabbit Abs. Because some of the fragments were dimerized by means of a disulfide bond, the analysis was run under non-reducing conditions. For unknown reasons, the dimerization of the HVR-B fragment was incomplete. No blotting signal was observed with preimmune rabbit serum, demonstrating that the weak IgG Fc-binding reactivity of M1 did not influence the results (not shown).

B. Similar analysis in the M5 system. Each set of blotting data is representative of two independent experiments.

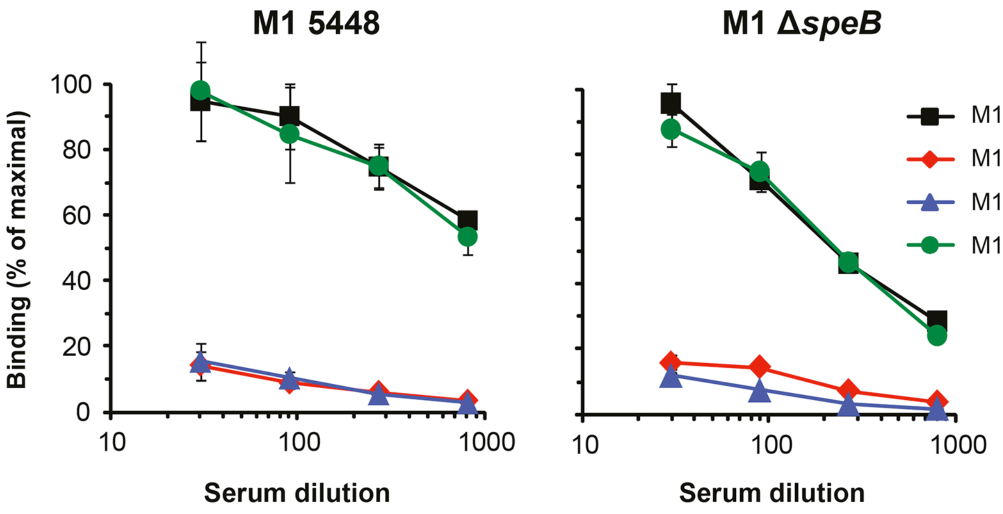

Fig. S3. Absence of SpeB does not affect the Ab response to M1 during experimental infection. The sera analyzed here were from mice infected with a sublethal dose of the w.t. strain 5448 or its SpeB-negative mutant, as indicated. Analysis of Ab reactivity was performed as in Figure 2F. Each panel shows results obtained with serum from one mouse. For each type of infection, similar results were obtained with a second serum. Data show mean values ± SD; each analysis was performed three times.

M1-F:	ATAT**GGATCC**AACGGTGATGGTAATCCTAGG
M1B-dim-R2:	ATAT**GAATTC**TTATTAGCATTCTCTAGTAATCGTTTCTAACT
M1C-F	ATAT**GGATCC**ACGATTACTAGAGAACAAGAGATTAAT
M1-R	ATAT**GAATTC**TTATTAGTTTTCTTCTTTGCGTTTTACA
M5B-F	ATAT**GGATCC**AAACAGCAAGAGAGTAAAGAA
M5B-dim-R	ATAT**GAATTC**TTATTAGCATGCCTCATCTTTTTTAGCTAA
M5C-F	ATAT**GGATCC**GCTAAAAAAGATGAGGCAAACAA
M5-R	ATAT**GAATTC**TTATTAATTTTCTTCTTTGCGTTTTACAA

Table S1. Primers used for PCR amplification. The underlined nucleotide sequences hybridize to the target genes. Endonuclease cleavage sites are indicated in bold. BamHI and EcoRI were used to insert fragments into the pGEX-6P-2 vector. Forward-primers are designated M1-F, etc. Some of the reverse primers are labeled “dim”, to indicate that they introduced a carboxy-terminal cysteine residue, used for dimerization.
